# Editorial: Factors affecting boar sperm preservation and quality

**DOI:** 10.3389/fvets.2023.1218940

**Published:** 2023-07-04

**Authors:** Elisabeth Pinart, Jane M. Morrell

**Affiliations:** ^1^Biotechnology of Animal and Human Reproduction (TechnoSperm), Institute of Food and Agricultural Technology, University of Girona, Girona, Spain; ^2^Unit of Cell Biology, Department of Biology, Faculty of Sciences, University of Girona, Girona, Spain; ^3^Department of Clinical Sciences, Swedish University of Agricultural Sciences, Uppsala, Sweden

**Keywords:** refrigeration temperature, L-leucine supplementation, fat-soluble vitamins, proteasome inhibitor, caffeic acid phenethyl ester (CAPE)

In the porcine industry, artificial insemination (AI) is performed using either refrigerated or cryopreserved seminal doses from selected boars. In AI programs, threshold values for sperm concentration, sperm viability, sperm motility, and sperm morphology are usually established to obtain high quality seminal doses with high fertility and prolificacy rates (1, 2). Reproductive performance of boars depends on genetic or intrinsic factors, such as breed, age or testicular size, environmental factors, mainly temperature and photoperiod, and husbandry factors, such as semen collection rhythm, diet, and social environment (Calderón-Calderón et al.). This Research Topic includes original research focused on the effects of supplementation with L-leucine (Leu; Lin et al.) and with fat-solube vitamins (Calderón-Calderón et al.) on male reproductive performance, the protective role of caffeic acid phenethyl ester (CAPE; Lan et al.) and temperature effects (Henning et al.) on sperm quality of liquid-stored semen doses, and the effect of proteasome inhibitor MG-132 on *in vitro* capacitation of boar sperm cells (Hackerova et al.).

In their comprehensive study, Lin et al. demonstrated that dietary supplementation with Leu from weaning to 10 months favors the testicular development and improves the semen quality of boars. This effect was mainly due to an increased expression of specific genes implicated in cell metabolism and cell proliferation, including those encoding for the mitochondrial branched chain amino acid transaminase (BCATm), cyclin B1 (Cyclimb1), serine/threonine kinase (AKT), as well as the mammalian target of rapamycin (mTOR). Notably, the expression of genes related to hormone synthesis, such as the cytochrome P450 enzyme (CYP19A) and the mitochondrial enzyme P450scc, and the synthesis of androgen receptor (AR) did not differ between supplemented and control males. In their complementary study Calderón-Calderón et al. focused on the effects of intramuscular administration of fat-soluble vitamins for 32 weeks on seminal parameters. The results clearly demonstrate that fat-soluble vitamin supplementation improves the semen quality of boars, resulting in an increase in seminal volume and in total and progressive sperm motility. Interestingly, morphometric sperm variables were also improved, indicating that fat-soluble vitamin supplementation influences spermatogenesis and epididymal maturation.

Nowadays, a wide range of commercial extenders have been developed for liquid preservation of boar semen under refrigeration conditions for short (3–4 days), medium (5–6 days), long (7–9 days), and extra-long (>8 days) term periods. Boar sperm is very sensitive to low temperatures, so the conventional storage temperature is currently at 17°C (Henning et al.). This temperature preserves sperm quality and fertility, but it also favors bacterial proliferation during storage, resulting in decreased sperm viability, motility, and fertility rates. Due to increased antimicrobial resistance, refrigeration at lower temperatures could be a reliable method to inhibit bacterial proliferation in liquid-stored extended semen doses. In their comprehensive approach, Henning et al. analyzed the effect of different storage temperatures (25, 17, 10, and 5°C) and times (24, 72, and 120 h) on sperm quality, metabolism, and energy status in seminal doses diluted in Beltsville Thawing Solution (BTS). The results obtained demonstrate that for storage periods ≤ 120 h (5 days) the optimal temperature ranges between 15 and 25°C; nevertheless, it should be remembered that these temperatures favor bacterial proliferation. On the other hand, storage at 10 and 5°C alters the ATP and nucleotide content, energy charge, acrosome integrity, and sperm motility and kinematics. These effects are dependent on the refrigeration time. Interestingly, refrigeration temperature (10 and 5°C) also impairs the energy status of sperm cells after incubation at 38°C. These findings must be taken in consideration in the formulation of extenders intended for the storage of liquid boar seminal doses below 17°C.

On the other hand, long-time liquid preservation of boar semen leads the accumulation of associated free radicals and reactive oxygen species (ROS) which results in oxidative damage of sperm cells. Lan et al. conducted a set of elegant experiments to analyse the protective role of caffeic acid phenethyl ester (CAPE) in liquid-stored boar semen. The supplementation of seminal doses diluted in Beltsville Thawing Solution (BTS) and stored at 17°C for 5 days with 210 μmol/L of CAPE led to a significantly higher total and progressive sperm motility and plasma membrane and acrosome integrity compared with control samples. Even under oxidative stress conditions and long-term storage (9 days), CAPE supplementation preserves sperm motility and mitochondrial activity, as well as the enzymatic activity of adenosine-monophosphate (AMP)-activated protein kinase (AMPK), glutathione peroxidase (GSH-Px), superoxide dismutase (SOD), and catalase (CAT). The protective role of CAPE was correlated with the activation of AMPK, which further increases the activity of GSH-Px, SOD, and CAT.

Sperm cells acquire their fertilizing ability in the oviduct, due to physiological, structural, and molecular changes called as sperm capacitation. These changes include tyrosine phosphorylation through the protein kinase A (PKA) pathway, increased calcium and bicarbonate inner levels, increased plasma membrane permeability, intracellular alkalinization, hyperactivation of sperm movement, and plasma membrane hyperpolarization (3). The increase in permeability requires the removal of decapacitation factors adhered to the plasma membrane of freshly ejaculated sperm, which is regulated by the ubiquitin-proteasome system (UPS) (Hackerova et al.). In their excellent approach, Hackerova et al. found that the inhibition of UPS system with the proteasomal inhibitor MG-132 does not affect the sperm viability, the pattern of tyrosine phosphorylation or the ubiquitination level during *in vitro* sperm capacitation; however, it suppresses total and progressive sperm motility and hypermotility in a dose-dependent manner (10–100 μM). These results agree with previous studies that demonstrated that UPS inhibition results in PKA dysregulation (5, 6) and defective degradation of A-kinase anchoring protein 3 (AKAP3) present in the fibrous sheet of the flagellum during sperm capacitation (4). This provides relevant clues about the process of boar sperm capacitation that may be used in the formulation of semen extenders and cryoprotectants to prevent premature capacitation.

## Author contributions

EP wrote the editorial article. JM reviewed and approved the document.
